# Announcements

**Published:** 1856-08

**Authors:** 


### ANNOUNCEMENTS.

Our table is covered with announcements, all of which indicate a flourishing condition of the several institutions which have honored us with their pronunciamentos. We are much gratified to observe that from certain indications, each is striving to excel in adding such additions and instituting such modifications and improvements as will redound to the advancement of their classes.

We have before us the annual announcement of the "Medical College of the State of Missouri," "New York Medical College," "Medical College of Ohio," "McGill College," Montreal, Canada, "College of Physicians and Surgeons," New York City, "Ruch Medical College," Chicago, Ill., and of the "Medical Department of the Pennsylvania College." Since the issue of the circular of this last institution, a notice of which will be found in our advertising pages, a change has taken place in the Faculty, by the resignation of Dr. J. M. Allen, who filled the Chair of Anatomy in that institution, and to supply the vacancy, Dr. T. G. Richardson, of Louisville, Ky., has been chosen. Dr. R. is, as is well known, the author of a work on Anatomy, and a gentleman long connected with the schools of that city. This selection was not only fortunate for the institution in which he is to labor, but is a compliment alike worthily bestowed upon Dr. R., and the profession of Louisville. It would appear that so soon as a vacancy occurs in the different institutions of the country, all eyes are turned to old Kentucky, and in an especial manner, to the cities of Louisville and Lexington. Dr. Gross has been but recently appointed to the place made vacant by the resignation of Prof. Mutter. A deep regret was everywhere felt by the profession of the whole country, that Prof. Mutter has withdrawn from that field of labor, but it is very much modified by the consideration that one so able has been chosen in his place. A regret is also felt that Dr. Allen has seen cause to leave the corps of teachers, as he is a most eloquent lecturer and successful teacher. Dr. R., however, will fill the void. Louisville has sustained a loss, but there is consolation in the reflection that she has not lost *all*.

While on this subject, we would remark upon the pleasure it has afforded us to observe that the medical profession keeps itself aloof from the errors of sectionalism. This shows great liberality on the part of the profession of the "city of brotherly love;" that they know no *North* nor *South*, in the profession, and the readiness with which these positions have been accepted, exhibits none of that narrow prejudice which characterises the actions and movements of men in some other departments. Science is universal, and its devotees are scattered every where over the country, evolving and progressing, irrespective of cardinal influences.

### THE ENSUING COLLEGE SESSION.

From present appearances, the next session which, as it will be seen by the announcement, will open on the 31st of October next, will prove to be even more largely attended than the last. Every mail brings us notices of intention, and inquiries in relation to the time of opening, and other information on the part of those desirous of attending.

It will be seen that the Faculty has been strengthened in a material point by an able Anatomist, and an old experienced teacher; but other changes are in contemplation, or rather additions made which will make, we hope, the next session a most profitable one to the student.

There are a few considerations which we would present to the student. One is, that we hope no one will present himself, unless it is his ardent desire to advance and improve. The Faculty will omit nothing in their power to promote this object. But they desire the attention of each member of the class, and therefore, if possible, each one should present himself at the opening of the session. After this he should suffer no interruption in his course of studies; and therefore, the student should come prepared to remain until the close. No vacation for the hollidays, except a day for each, nor any other for any purpose until the close of the session. We make this statement now that those having determined to be present may make their arrangements before leaving their homes.

We desire an attentive and studious class, rather than a large one, made up of those who come, passively, to be instructed, without an effort on their part, while the time which should be devoted to study is spent by them at the theatre, or other public resort. Such will neither improve themselves nor permit others to advance, who may desire to do so. We would take this occasion to say that we *cannot* approve of such conduct.

Fortunately for us, we have been without these annoyances and these sources of mortification, except in a very few instances. Our classes have been such, in the main, as were objects of pride with the teachers, who labored the more ardently for their good, because they saw a desire to improve.

